# Revisiting Epigenetics Fundamentals and Its Biomedical Implications

**DOI:** 10.3390/ijms25147927

**Published:** 2024-07-19

**Authors:** Thuluz Meza-Menchaca, Arnulfo Albores-Medina, Alma Jaqueline Heredia-Mendez, Eliel Ruíz-May, Jorge Ricaño-Rodríguez, Verónica Gallegos-García, Adriana Esquivel, Giancarlo Vettoretti-Maldonado, Alma D. Campos-Parra

**Affiliations:** 1 Laboratorio de Investigación en Ciencias Médico-Biológicas, Facultad de Medicina, Universidad Veracruzana, Médicos y Odontólogos s/n, Col. Unidad del Bosque, Xalapa 91010, Mexico; zs23000353@estudiantes.uv.mx (A.J.H.-M.); aesquivel@uv.mx (A.E.); zs21006843@estudiantes.uv.mx (G.V.-M.); 2 Departamento de Toxicología, Centro de Investigación y de Estudios Avanzados del Instituto Politécnico Nacional, Ciudad de México 07360, Mexico; aalbores@cinvestav.mx; 3 Red de Estudios Moleculares Avanzados, Cluster BioMimic®, Instituto de Ecología A. C., Carretera Antigua a Coatepec 351, Congregación el Haya, Xalapa 91073, Mexico; eliel.ruiz@inecol.mx; 4 Centro de Eco-Alfabetización y Diálogo de Saberes, Universidad Veracruzana, Zona Universitaria, Xalapa 91090, Mexico; jricano@uv.mx; 5 Facultad de Enfermería y Nutrición, Universidad Autónoma de San Luis Potosí, San Luis Potosí 78210, Mexico; veronica.gallegos@uaslp.mx; 6 Instituto de Salud Pública, Universidad Veracruzana, Xalapa 91190, Mexico

**Keywords:** epigenetics, epistemology, biomedicine, omics

## Abstract

In light of the post-genomic era, epigenetics brings about an opportunity to better understand how the molecular machinery works and is led by a complex dynamic set of mechanisms, often intricate and complementary in many aspects. In particular, epigenetics links developmental biology and genetics, as well as many other areas of knowledge. The present work highlights substantial scopes and relevant discoveries related to the development of the term from its first notions. To our understanding, the concept of epigenetics needs to be revisited, as it is one of the most relevant and multifaceted terms in human knowledge. To redirect future novel experimental or theoretical efforts, it is crucial to compile all significant issues that could impact human and ecological benefit in the most precise and accurate manner. In this paper, the reader can find one of the widest compilations of the landmarks and epistemic considerations of the knowledge of epigenetics across the history of biology from the earliest epigenetic formulation to genetic determinism until the present. In the present work, we link the current body of knowledge and earlier pre-genomic concepts in order to propose a new definition of epigenetics that is faithful to its regulatory nature.

## 1. Ancestry of Epigenetic Concept

Since the earliest biological concepts, the essential definition of genetics focuses on the heritage of descendants from generation to generation with a specific structure unit built by Deoxyribonucleic Acid (DNA), called a gene. The Danish botanist Wilhelm Johannsen, who first coined the word “gene” (from the Danish/German “gen”) at the beginning of the XX century, helped to afterwards describe it as hereditable traits in a genomic identifiable structure with a proven biological function [1]. Nevertheless, at that time, Johannsen’s ‘gene’ did not provide the entire answer to relate any given biological function to its particular structure. He stated that the gene ‘Was nothing but a very applicable little word’ [2]. Although Gregor Mendel’s theory was developed at the time, he mentioned that ‘The Mendelian units as such, taken per se are powerless’ [3], and the genius work from Gregor Mendel was ignored for decades until Theodor Sutton and Walter Bovery proposed the chromosomic theory [4,5]. In that context, it took some time to define the role and composition of genes and what we now know as Mendelian disorders of epigenetic machinery [6].

Comparing what William Johannsen visualized as a gene and the coining of other very common names in the genetics field, such as genotype and phenotype, all related to an observable appearance of plant pigmentation, has now become a far more intricate concept. The scientific understanding of inheritance has improved as a component in the survival of species, as well as in evolution, and from health to disease.

Overall, the literature on genetics has averred succinctly, not only as a conserved structure element but also as how it varies and diversifies and how either of these two could affect the function that can occur within the cell, tissue, or organ systems. Therefore, the result of the simplest observable trait involves more than a simple gene to produce any identifiable role, different from originally expected, whether it is during developmental growth, differentiation, or post-developmental regulative growth control. Also, across the life span of species, time-restricted or space-specific cellular behaviors as a set of normal as well as abnormal patterns may occur in a disease that is heritable or not, or in even infection proliferation or, rather, in the provision of immunity to the same disease. 

From its remote origin and before the dawn of the concept of epigenetics, the binomial terms intersected by genetics and environment were concisely built over genetic determinism [7]. Two of them, epigenesis and preformism, both terms alluding to how a whole being starts from a miniaturized cell, were present in the 17th and 19th centuries [8]. Interestingly, a more recent study showed that young adults believe in genetic determinism [9]. However, epigenetics’ conceptual roots go back further to the remote past as epigenetic inheritance and germline reprogramming were parallel concepts that had overlapped meanings. This reprogramming enables the totipotential zygote, and as a paradigm of developmental biology, the term “epigenesis” was proposed by Aristotle in his book: *On the Generation of Animals* (384-322 BC) [10]. This theory opposed the preformationism hypothesis by Anaxagoras and Hippocrates about a century and a half earlier and was created by the systematic analysis of chicks’ embryos throughout development in his pioneer works as perhaps the first developmental biologist. However, it was not until the XVII century that it became possible to track back the word epigenetics to the Renaissance. In a book centuries later, the word re-emerged in the works of the physician and physiologist William Harvey in 1651 and 1653, Exercitationes de generatione animalium and Anatomical Exercitations, that were followed by Aristotle’s experiments with chick embryos, explaining that the concept was the “additament of parts budding one out of another”. This work lead to the discovery of the circulatory system deduced for humans from the avian model [11]. 

Contemporarily, with the earliest descriptions of a cell from magnified images through microscope lenses, Harvey did deny those observations based on a very closely supported view of Aristotle. Harvey proposed a new theory of generation called epigenesis when he published the Generation of Living Creatures. This new paradigm contrasted with the existing one of “metamorphosis”, in which it was believed that development happened all at one time. On the other hand, through Harvey’s observations of how particular organs, such as the heart, were formed before the rest, and how blood was that important for the beginning of life, we sustained a more reliable sense of embryogenic development as a systematic process. His contemporary René Descartes was skeptical from a purely mechanistic view, one of the greatest intellects of mankind. 

Descartes, for the concept of biomedical function, visualized an organism body analogous to machines that could adapt one part to another one [12]. This kind of scope was not well received among the ecclesiastic quorum because it denied the preformed developed structure right after embryogenic conception. Parallel at that time, René Descartes´ epigenesis concept was followed in the XII and XIII centuries by Pierre-Louis Moreau de Maupertuis who was convinced of the epigenetic hypothesis and opposed to preformism. Maupertius Newtonian observations´ scope concluded the improbable formation of a perfect and functional embryo immediately after a simple random collision of organic particles. These arguments were a combination of Newton’s gravitational pull and the recent discovery of “relationships”, or chemical affinities, by the French naturalist Étienne François Geoffroy (1672–1731) [13]. In this sense, Maupertuis proposed the existence of hereditary particles [14].

In contrast to de Maupertuis, a slightly different modification was proposed by George-Louis Leclerc, Comte de Buffon, who explained epigenetics as enumerating three different variables: first, organic matter as all living substances; second, an inductive force that “pushes or attracts the organic parts of the nutrient”, which could be interpreted as biomolecular charges; and a third internal mold that could be interpreted as the genetic code or DNA with instructions that shapes a particular form of matter (moule interieur´) [15].

Across the eighteenth century, the naturalist John Turberville Needham, supported by microscope observations, described the independent generation of cells. Tuberville, among the materialists in the same century such as Julien Offray de La Mettrie, Denis Diderot, and Paul Henri Thiry D’Holbach, supported the mechanistic side of knowledge put forwards by Newton’s thought in terms of the self-organizing development of organisms, applying the attractions and repulsion of the Newtonian forces [16].

On the other hand, the vitalistic view of supporters like Caspar Friedrich Wolff and Johann Friedrich Blumenbach caused a change of paradigm from the mechanistic to the vitalistic [17]. The vitalistic view had new aspects to back up its thought on epigenetic powers such as spontaneity, irritability, sensitivity, and intelligibility. In a book called Theoria Generationis, this concept is applied by visualizing the phenomena of development as a progressive change that rises in complexity [17]. This view as a center of origin contrasts with preformation, which proposed a “supernatural epigenetic” role where development was only a growth in size starting from a miniature version of any given organism, as Charles Bonnet claimed [18].

According to Immanuel Kant, organisms are not formed by occasional miracles (occasionalism), nor from the preformation of the immediate ancestor. Instead, Kant had a different idea about the growth model, through aggregation, where epigenetic formation is progeny, in which predecessors have internal functional purposes (inneren zweckmäßigen Anlagen); this was called generatio homonyma as a phylogenetic mechanism proposed by Kant [19]. In this sense, he assumed that species evolved from simpler ones. However, less complex species are not created from unorganized matter. Nevertheless, this did not represent a scientific theory of evolution because he believed that the force of evolution was subjected to morality under God’s creation of life and all nature. And it was not until early in the XIX century that the first theory of evolution was presented by Jean Baptiste Lamarck (1809). In his book Philosophie Zoologique, he proposed that the environment creates needs and unorganized matter can produce simple organisms that can evolve into a more complex one under a logic of use, inheriting morphological features, and of misuse, comprising erasing a given feature in the progeny [20]. In opposition to this, a biologist, Hans Driesch, stated that Lamarck presented a fictitious construction with no evidence [21]. 

Unlike Jean-Baptiste P. A. de Monet Lamarck, Alfred Russel Wallace and Charles Darwin had empiricist approaches to verify hypotheses through observations, and together, they developed the natural selection theory and explained how it could affect evolution. As well as Kant and Lamarck, Charles Darwin thought that the environmental input was significant enough to produce an effect on species in terms of any observable adaptative phenotypic variation at an intrapopulation spot. Darwin named the heritable molecules “gemmules” [22]. Darwin called this scope of inheritance the pangenesis theory, which adopted the Lamarckian mechanism of evolution involving use and misuse. In contrast, this was disproved by Mendelian genetics, which to some extent validated the genetic traits of heritage segregation. However, there is written evidence of the acceptance of the adaptation theory found in observing species of Hieracium. On the other hand, he ignored any precise mechanism of how heritage is transmitted, sending information from somatic cells to the germ line from generation to generation [22]. 

For instance, the clear phenotypical polymorphism in ants provides evidence that there is something more than just an exact expression from progenitors to descendant generations [23]. According to Kant, organisms do not grow simply by adding more material but instead are defined as an output from the environment and defined as a transformant principle for differentiation and growth.

In phenotypical biodiversity even in the same species, between castes, there is a clear correlation of eusocial species and labor-specific traits [24]. Under Darwin’s statements of how selective pressure might be a force of natural selection and evolution, it might have been a more complex task to explain how diverse phenotypes in the same species were, and how these might have played a specific biosocial role. These conclusions have been revisited by Darwin’s observations, who inferred that the evolution of morphophysiological relatives divided them into exclusive selves partitioning all reproductive tasks or, on the other hand, numerous sterile descendants related to providing food. This contrasted to Darwin’s theory that single individual forces oversee the success or failure of segregating phenotypical traits among the next generation.

In this sense, Darwin faced a difficulty: the great difficulty lay in the male and fertile female working ants differing greatly in structure, such as in the shape of the thorax and in being destitute of wings and sometimes of eyes, and in instinct. As far as instinct alone is concerned, the wonderful difference in this respect between the workers and the perfect females would have been better exemplified by the hive bee. Under these circumstances, compiling evidence has occurred over the last decades, unveiling new mechanisms in epigenetics linked to the environment of insects [25].

Nevertheless, this phenomenon where the balance between morphologically distinct worker castes depends epigenetically on the levels of histone acetylation was found in social insects like the carpenter ant *Camponotus floridanus*. There is another type of environmentally induced chromatin regulation. Interestingly, it can be influenced by feeding behavior [26].

Genetics, at the level of heredity, has advanced not only in terms of DNA but also its expression and the environment’s influence. Interestingly, the work of Lamarck, Darwin, and Wallace has been revisited until the present day, and although the right mechanisms were waiting to be uncovered, the ecological influence was often considered. Now, the phenotypical traits result in several mechanisms of edition in DNA, which we will revise in depth in the following sections [27].

## 2. Contemporary Epigenetic Scopes

A broad spectrum of conceptual epigenetic terms has been advertised throughout our entire history, starting with the Aristotelian theories until today, as a continuous process of transformation. Especially more contemporary ones, researchers and studiers may agree on one term or another, depending basically on the field of study [28]. Epigenetics´ concepts in history can be placed in different epistemological, methodological, and metaphysical approaches based on the guide of a particular field and the approach of the scientific investigation in which it is involved. Nevertheless, perhaps one of the most adopted concepts in the scientific community is that epigenetics is related to any stable and heritable change in a cellular phenotype or genetic profile that has the same DNA code [29]; however, to obtain to this term, a lot of knowledge has been gained.

The term epigenetics was coined and also defined by Conrad H. Waddington in the late 1930s. The term was taken from Greek; he stated it as “the study of all the events that lead to the development of the genetic program of development” [30] or through the complex “developmental process that mediates between genotype and phenotype” [31]. Since the origin of the concept of epigenetics, established by the Scottish embryologist Waddington, he worked in contemporary biology in the 30s and 40s, looking into embryogenic development. And by looking at how the fertilized egg progressively becomes a fully grown mature organism, he discovered that it centers on how the regulation of gene expression enables the cellular differentiation of isogenic cells. Waddington’s main focus was to understand the development of a system in which broad interactions may occur within interactions of several cell lineages [30]. He states that the different layers of mechanisms at the cellular and molecular level cannot be caused by a single gene or biomolecule. As a following exponent and part of its tradition, David Ledbetter Nanney´s work, genetic “*Controls Systems*”, emphasized the developmental process as a mainstream of a multitude of molecular factors and, on the other hand, denied that only DNA and histone methylation had the exclusive role of controlling cell gene expression. Waddington conceived them as developmental constraints and proposed that epigenetics are present in cell-to-cell communication as well as at the molecular level in a web that altogether could organize a specific phenotype’s visible trait. Therefore, a complex net of interactions among biomolecular and cellular levels and even the environment was hypothesized by Waddington over what he called an epigenetic landscape under an ontological view [31]. Under Waddington’s scope, epigenetics could be considered a type of epigenesis in which development is a complex interaction between biomolecules and the environment; in fact, the term epigenetic landscape is defined as a platform for the interaction of a variety of environmental factors with a complex genetic environment to change the expression of genes [29,30,31]. Due to this hypothesis, it is not possible to see the epigenetic landscape solely as “single gene centered”, but at the same time, this cannot be discarded. Therefore, at the beginning of the 20th century, genetics was considered the science of heredity, and embryology was considered the science of development [32], two divided and separated fields. Waddington tried to demonstrate that both disciplines were closely linked to each other and to evolution, so that the explanation of development from genotype to phenotype would necessarily have to integrate the knowledge of both sciences.

Interestingly, Waddington’s hypothesis did not contain any information about where exactly a gene was located in the cell, and a couple of years later, Oswald Avery, Martha Chase, and Maclyn McCloud, by retaking experiments from Frederick Griffith, found out that DNA was the hereditary material [33]. Following on from the approach of Waddington’s thesis, through a different perspective of epigenetics, DNA has something above, at the top, or upon genetics, thus epigenetics. 

A clear historical landmark in molecular biology was the modeling of the DNA structure performed by James D. Watson, Francis Crick (1953) [34]. This led to a gene-centered view of genetic determinism that would permeate the rest of the XX century as the pre-genomic era, where this approximation was well revisited by Herman A. Burbano (2006) [7].

This conceptual statement was coined on the epigenetic control systems (1958), whose role is to “drive” according to the information that is encoded in the DNA. In this context, the microbiologist David Nanney, in 1958, studying biochemical events such as environmental effects on enzyme synthesis, had a rather different view of the meaning of epigenetics. The microbiologist divided cell control into two separate systems, one based on DNA sequences and a different one based on determining which were expressed in a particular cell, called the “auxiliary mechanisms”, which are now known as the control expression machinery [35].

Addressing the Waddington epigenetics report (1942) as a lower level of importance in development and highlighting the genetic systems [31], these observations might have been based on the achievements by Joshua Lederberg of the characterization of the transcriptional machinery [35]. The microbiologist Lederberg made significant contributions to the biomedical field by using *Salmonella* ssp. as a model. However, this scope solely used histone modification or DNA methylation to affect a given phenotype of a particular cell [36]. It was not until nearly half a century after Waddington had opened Pandora’s box that a major contribution pointed towards separating two very important steps, the sexual recombination of inheritance and developmental growth in adulthood. This fact is considered Robin Holliday’s main contribution to identifying the classic mechanism of chromosomal recombination and called the chromosomal Holliday junction [37].

From the pioneering term of epigenetics, referring not specifically to any molecular cascade or pathway that might have been involved in changing a given phenotypic trait, after more than 50 years of growing evidence in the molecular biology field, the generally accepted concept has obtained a more accurate definition as specifying mitotically and/or meiotically heritance and one that does not entail a change in genomic DNA. The most widely accepted theory is that epigenetics can be defined as a post-replication event due to alterations in the gene expression patterns in a single cell lineage absent from changes in the genomic DNA sequence. In a different scope, and rather more recently defined, a matter of open debate is whether a given mutation is confined to a somatic cell in a particular tissue, and therefore is or has not been able to pass from one generation to its descendants. In this sense, the two-century-old ideas of the inheritance of acquired traits and segregation have been revived. This scenario includes the view of Jean Baptiste Lamark, which clearly disrupts Gregor Mendel’s postulates of segregation traits [38]. This phenomenon was first discovered in yeast, fruit flies, and plants, and was described convincingly in mammals in 1999 by Emma Withelaw and her collaborators using a genomically almost identical strain of mice with a slight difference in coat color from mottled to yellow. The yellow mother had more yellow pups and the mottled mother had more mottled pups; the epigenetic marks introduced enabled the segregation of their characters.

…“*The work has quite correctly taken on a “Lamarckian” flavour and is quite frankly another milestone in the paradigm shift now underway in molecular and cell biology, which is now impacting on a clearly observable trait, regulation of coat colour*”….

[39]

Therefore, this is a rather different view from the traditional perspective of how changes that cause variation in expression are induced by mutations, such as variations or changes in the DNA. Those epigenetic mutations take place in genes that are silenced and can be abnormally activated, or the opposite occurs, where other genes are engaged in epigenetic regulation, which has an effect on a transcriptionally repressed RNA that, since it is normally active, can produce an observable aberrant phenotype, for example in development. In both cases, abnormalities can be easily observed; subtle phenotypic changes, caused by epimutations, might be difficult to screen and detect. However, that does not mean it is not possible for them to happen. 

Accordingly, several different epigenetic epistemic traditions define how the outcomes of the changes in expression patterns occur or have been produced:

(A) As described earlier, Conrad Waddington was the first to describe the concept of epigenetics in the early 1940s, as “the branch of biology, which studies the causal interactions between genes and their products which bring the phenotype into being” [31].
(B) Robin Holliday stated in 1990 that there is temporal and spatial control of gene activity during the development of multicellular organisms. From this point of view, whether mitotically and/or meiotically, heritable diverse gene functions, without a specific origin, are not related to changes in the DNA sequence [37,38].
(C) Bartolomei in 1991, found diverted expression (imprinting) although DNA sequence remains constant in mice [40].
(D) Epigenetic modification occurs from ncRNAs [41].
(E) Through its mechanism, generally, epigenetics is determined by modifications in gene expression independent from the DNA sequence: “the study of changes in gene function that are mitotically and/or meiotically heritable, and that do not entail a change in DNA sequence” [41].
(F) They are the result of potentially inheritable epimutations [42,43].

**Figure 1:**
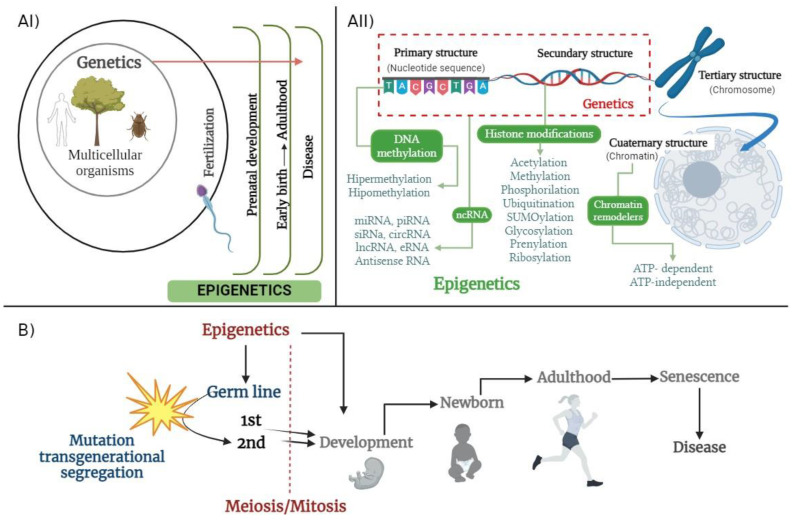
(**AI**) Growing phenomena of epigenetic spectra spotted across lifetime versus genetics as central control of information in multicellular organisms; (**AII**) correlation of genetics and epigenetics understood under molecular structural view; (**B**) epigenetics in life span across inherited generations and parallel cell division.

The compiled amount of evidence has contrasting explanations in terms of epigenetic variance through inheritance [41,43,44], restricting the phenomena exclusively to paternal contribution (Figure 1(AI)) [45,46,47] and other subsequent related mechanisms, such as environmental factors [43,45,48]. Additionally, transgenerational epigenetic stability [44,49,50] has been defined differently, from intergenerational epigenetic inheritance, which represents the direct heritage of epigenetic marks from one generation to the next, which means directly from parents to children, to transgenerational inheritance, which has been defined as descending from F0 to F2, in other words, from grandparents to grandchildren [43,44,50]. An epigenetic event was set up in F0, depending on if the mother or the father had exposure to the event. In the case of the father, the epigenetic inheritance would have started right through F1. Conversely, if it were to happen to a pregnant mother, it may occur differently. The classical concept of transgenerational epigenetic inheritance has an effect until the F3 generation due to the fact that if the event happened in the pregnant mother, it would affect the child, but also their own germline. Therefore, in the following consecutive generations, an epigenetic effect from F2 and further onwards would occur. In this sense, Jackson and collaborators (2013) proposed a comprehensive explanation [51]. This, however, did not explain how the grandmother’s ovary may contain the instructions for the mother and therefore the granddaughter and how after meiosis, many epigenetic regulatory events are present in human stages throughout life span (Figure 1B). 

## 3. Epigenetic Biomolecular Mechanisms

Molecular functional roles can be modulated through various epigenetic mechanisms, which are processes that can be reversible, in comparison with genetic mechanisms of gene modulation. Mechanisms can either have a tag, for instance in methylation or histone modifications, or RNAs can interfere with what exactly is stated in the DNA code (genetics) in terms of epigenetic mechanisms, as illustrated in the following examples.

### 3.1. Methylation

A major mechanism in silencing gene expression is methylation (Figure 1(AII)) and the number of methylations or demethylations can affect gene expression; this is commonly referred to as hypomethylation and hypermethylation, respectively. The potential capacity to generate novel routes with DNA methylation, due to environmental variation, for phenotypic diversity is tightly linked with DNA methylation in adaptive evolution [52]. DNA methylation is involved in phenotypic inheritance in single-celled organisms, as well as in transgenerational inheritance in multicellular organisms. This mechanism consists of the covalent addition of a methyl group (-_CH3_) to carbon five of the pyrimidine ring of cytosine, resulting in 5-methylcytosine (5 mC) [53]. In mammalian somatic cells, this modification occurs predominantly in 98% of 5’CpG3’ dinucleotides; on the other hand, in embryonic stem cells, only 75% of CpG dinucleotides are methylated. However, there is evidence that the methylation of DNA can occur in non-CpG dinucleotides, such as CpA, CpT, or CpC [53]. In a mammalian genome, the distribution of CpG dinucleotides is random, exhibiting regions of low and high density, known as CpG islands [52]. Interestingly, in the human genome, between 60% and 80% of the total CpG dinucleotide content (29 million sites) is methylated, and these sites are associated with repetitive sequences, transposable elements, and tissue-specific gene promoters. On the other hand, 70% of the promoters of the total protein-coding genes are associated with CpG islands, and most of these sequences are demethylated [54]. DNA methylation plays an important role in different molecular mechanisms, such as the regulation of gene expression, where the methylation of CpG islands and promoters is a recognition platform for different transcription factors or proteins that recruit different factors. Also, it can remodel the chromatin structure and inhibit transcription and subsequent gene expression [53]. However, the methylation of the gene body appears to contribute to elongation during transcription, the regulation of alternative splicing, and the regulation of non-coding RNAs. In addition, DNA methylation is highly relevant during X chromosome inactivation, genomic imprinting, chromosome stability maintenance, and the suppression of repetitive elements in the genome [53]. Given its reversible nature, DNA methylation is regulated by DNA methyltransferases (DNMTs), which catalyze the transfer of a methyl group from a donor group called *S*-adenosylmethionine (SAM) to cytokines. In mammals, DNMT1 has been identified as de novo methyltransferase, whereas DNMT3A and DNMT3B are maintenance methyltransferases. On the other hand, a group of enzymes called TET (10–11 translocases), which are monooxygenases, mediate the sequential oxidation of 5mC, and together with single-base excision, DNA repair machinery, and thymine DNA, glycosylase (TDG) demethylases DNA [55].

### 3.2. Histone Modifications

For the case of histone modifications, even in prokaryotes, there is evidence of histones H2A and H4 being responsible for the early emergence of a broad occurring structure in eukaryotes [56]. In human cells, as in the vast majority of eukaryotes, the DNA is condensed in the nucleus as chromatin. The first level of chromatin organization is known as the nucleosome, and it is composed of two copies of histones, which are the core protein components of the chromatin complex. Histones H2A, H2B, H3, and H4 are assembled in the nucleus, as an octamer core surrounded by DNA. There is an external histone to the octamer known as the linker histone or H1 that participates in the compaction of the nucleosomes [53].

Nucleosomes are unstable, and their structure changes rapidly in response to external stimuli, leading to permanent changes in the organization of chromosomes, thus exhibiting different domains, such as heterochromatin and euchromatin, that are defined by their degree of compaction and are associated with gene functionality [57]. The main mechanism by which the structure and the function of chromatin are regulated is through post-translational modifications in the amino acids of histone proteins, such as methylation, acetylation, ubiquitination, phosphorylation, SUMOylation, ribosylation, propionylation, butyrylation, crotonylation, and citrullination [58].

Of the above modifications, the most widely studied are acetylation and histone methylation. Acetylation occurs in the *N*-terminal lysine residues of histones, and this mechanism is mediated by acetyltransferases (HATs) that are responsible for adding acetyl groups to the tails of histones. On the other hand, the deacetylases remove acetyl groups of acetylated lysine. This modification in tail histones plays a very important role during transcription regulation through two mechanisms. Firstly, it refers to the ability to neutralize the positive charge of the lysine by adding the acetyl group to histones, thus forming an affinity between DNA (negatively charged) and histones less tightly arranged, and consequently, greater accessibility of DNA to transcription factors is observed, for example. And secondly, it is involved in the recognition of acetylation marks on histones by different proteins which can recruit different classes of chromatin structures, regulating proteins to promote or inhibit gene transcription [55,58].

Histone methylation occurs in lysine and arginine residues in histone tails, where histone methyltransferases transfer to a methyl group from a donor, SAM; demethylation is orchestrated by histone demethylases. Methylation or demethylation in histones results in the activation or inhibition of gene expression, respectively, by modulating DNA access to transcription factors through relaxation or compaction of the chromatin structure [55].

Post-translational modifications in the amino acids of the histone tails are recognized as the histone code, which can affect the accessibility of different proteins to the DNA. In this sense, the marks on the histone tails would be like a recognition signal for proteins and chromatin regulatory complexes. This code also has an impact on the structure of euchromatin and heterochromatin. In this way, the different combinations observed in histone tails can vary between nucleosomes, and the information contained in the code could be inherited by daughter cells after DNA replication [55]. The histone codes are involved in cell differentiation, cell mitosis, the repair of DNA damage, and X chromatin inactivation, and mediate transcriptional inhibition or activation, leading to many biological effects [55].

Over the past two decades, an increasing amount of information has provided evidence about transgenerational inheritance as a plausible mechanism in plants, due to asexual vegetative reproduction, but still, the fact remains that sexual gametes come from fully matured vegetates. Although less common in animals, inherited DNA methylation could also be present. In this sense, phenotypic changes can be environmentally induced and segregated across offspring from generations to generations; particular studies have been centered on the effect of stress in a wide range of model organisms [59].

Nevertheless, on the other hand, since the first discovery of the *Retinoblastoma tumor suppressor gene 1* (RB1), a transcriptional factor with demethylation capacities, if we consider that to date, more than 2000 transcription factors and 1600 in humans [60] have been characterized in cells, only a small number of them have been related with demethylation or methylation. In this manner, efforts must be directed towards screening the mechanism, perhaps combining methods comprising novel approaches with innovative systems that could identify how transcription factors involve epigenetic methylation [60]. 

On the other hand, to understand errors in methylation or the opposite, abnormal demethylation, it is necessary to question all of the other factors that occur in this mechanism, as perhaps the environmental input varies. This may provide a better approach to diagnoses, prognostics, and perhaps treatments, which could enable progress in understanding disease processes. This is due to the fact that, for instance, abnormal demethylation is present in several serious diseases, like cancers.

### 3.3. Interference RNA in Epigenetics

Interference mechanisms, mediated by non-coding RNAs (ncRNAs), include types which are classified according to their size, such as long non-coding RNAs (lncRNAs) and small non-coding RNAs (sncRNAs), such as microRNAs (miRNAs), small interfering RNA (siRNAs), circular RNAs (circRNAs), antisense RNAs, and piwi-interacting RNAs (piRNAs) [53]. These interactions between RNA and DNA can change gene expression without altering the DNA sequence, which remains constant. In this sense, its mechanism of action has been considered epigenetic.

Mature miRNAs are RNA molecules of around 20 nucleotides in length that are widely conserved among species with diverse functions and involved in the regulation of gene expression in multicellular eukaryotes and some unicellular eukaryotes. This type of ncRNA is derived from transcripts, intronic and intergenic sequences, that form a unique hairpin structure to be processed into their mature form, which allows them to mediate gene silencing at the post-transcriptional level by coupling to an RNA-induced silencing complex in the cytoplasm. Very interestingly, it is estimated that 30% of human genes are regulated by miRNAs [53,61].

lncRNAs are a different type of molecule of 200 or more nucleotides in length, and can originate from a sense or antisense form, depending on their proximity to the genes that encode proteins. These lncRNAs can be bidirectional, and they can come from intronic or intergenic sequences. They can also be derived from the disruption of open reading frames or chromosomal rearrangements. It is currently recognized that lncRNAs can serve as precursors of siRNAs; they can serve to modulate gene expression through the recruitment of chromatin-modifying enzymes, and they also serve as scaffolds for multiple proteins necessary for the assembly of ribonucleoprotein complexes, which can modulate the marks on histone tails [53,61].

### 3.4. Antisense Transcripts

The term referred to as an antisense transcript is an RNA molecule transcribed from the opposite strand of conventional (sense) genes. This kind of transcription has no precise direction from centromere to telomere; instead, it has a bidirectional expression. From the major regulatory epigenetic mechanisms, natural antisense transcripts (NATs) cover a broad and ubiquitous spectrum, besides complementary sequences indirectly interacting with other targets like DNA methyltransferases, histone acetylases, and deacetylases [62].

Once considered transcriptional noise, also similar to all of the non-coding RNAs, as the case of NATs’ weight of evidence has fast evolved throughout the years, they have become a significant regulator of gene expression. They display conservation all across the kingdoms of life, being involved in transcription or in ncRNA genesis. NATs are capable of being active in all stages of the transfer of information in the central dogma for transcription, translation, replication, sometimes conventionally named differently, and also RNA degradation. Their genomic structure enables the regulation of their expression in cis, but could also regulate expression in transposition, although it depends on how large the domain is. Either way, it could contribute to gene expression switching transcription, and also, it can act within protein/complexes as a modular scaffold; in both functions, it can rewire expression machinery [63].

NATs can either overlap or not with mature conventional sense sequences [62]. There are diverse types of ncRNAs, divergently expressed, that overlap with protein-coding genes and are also widespread in mammalian genomes. During its discovery, it was brought to our attention the fact that a considerable amount of the transcriptome was endogenous transcripts with inverse orientation serving as a template of RNA polymerase II [64]. Commonly, this kind of ncRNA transcript, in the opposite strand and reverse direction from the protein-coding RNA, is transcribed. Initially, they have been categorized within two different groups: On the one hand, there is the NAT group, which is complementary in situ with its sense counterpart, partially or completely. The second group is non-overlapping with the sense transcript, but still complementary to the DNA at specific gene boundaries. In the same manner, their sense transcripts can be imprinted, polyadenylated, and spliced; however, this event might become less common than the conventional sense and protein-coding pairs [65].

### 3.5. Special Cases: Piwi-Interacting RNAs (piRNAs)

First discovered in the *Drosophila melanogaster* germ line, piRNAs are part of the sncRNAs, which are involved at transcriptional and post-transcriptional levels [66]. Where other types of ncRNAs can naturally be classified in the same group due to structural features in common RNAs like miRNAs, endo-siRNAs, all of which fall into one category, which makes them particular, they have two things in common: the Argonaut partner and the distinguishable size of the mature transcript [67]. piRNAs regulate epigenetic mechanisms involved in germ cell development, and inaccurately, this kind of ncRNA has been only categorized in animals, while the correct definition is within the metazoans group [68,69]. Due to such a heterogeneous group of RNAs, it has received considerable attention among researchers over the last decade or two [70]. The lack of sequence homology and conserved structural motifs across species makes them very hard to characterize, in comparing different models of studies. Nevertheless, the proteins related to their mechanism remain unknown. piRNAs are somehow longer than miRNAs, being 21–35 nucleotides long, and can repress complementary transposons [70].

In theory, transposons could evade silencing through target site mutations, which reduce piRNA complementarity, so they are related to fighting viral infections. piRNAs lead PIWI proteins to degrade any specific target, recognized by the complementary RNA sequence, or even to methylate DNA, histone modification, or contribute to chromatin opening. Their mechanism of action allows for regulated conserved genes, but they are also capable of adaptively targeting to rapidly evolving viruses and exosomes. There is a close interaction between piRNAs and gametogenesis and development in metazoan. piRNAS are more expressed in germ cells than in somatic cells. Various reports show that the aberrant expression from piRNAS and PIWI proteins relates to a wide diverse type of human cancers; therefore, they have been proposed as markers in cancer clinical diagnosis [70].

### 3.6. Small Interference RNA

siRNA mediates in nuclear and cytoplasmic silencing pathways by triggering a permanent mark, which is potentially heritable in the gene, by silencing it in an epigenetic manner [71]. According to the emergence of the early discovery of siRNA, first found in plants, and afterwards in Platyhelminthes, and mammalian cells, a qualitative change was made in the scope of biomedical research. In the therapeutic context, in terms of a wide range of diseases, including cancer, with less effective drugs available, siRNA has been considered to have the potential to silence specific genes. DNA insertion strategies vary from viral to non-viral systems, from in vitro approaches to in vivo approaches, comprising lipids, polymers, or even peptides that can be inserted into the specific genome target to stop transcription [71].

As transporters, complex systems may have interactions called supramolecular complexes, which consist of aggregations of low-molecular-mass particles, and also large structures, which are assembled differently from those commonly described structures as covalently linked backbones [72]. These multimeric biomolecules can display functional capabilities during the delivery process.

### 3.7. Circular RNA

A recently described and not fully characterized type of exonic ncRNAs are covalently bonded from the 5′ to 3′ sticky ends in the shape of a circle, which is why they have been named circular RNAs (circRNAs) and are very commonly found in mammals. Although this group of RNAs comprises commonly endogenously expressed regulatory RNAs, recently, they have also been found to act in trans due to extracellular vesicles. circRNAs have a key role in the epigenetic mediation of gene expression at both transcriptional and post-transcriptional levels with a demonstrated function in liver diseases and homeostasis [73]. Apart from that, they are quite robustly expressed, well conserved in specific eukaryotes tissue, and developmentally stage-restricted. circRNA biogenesis has been described as back-splicing in the post-transcriptional stage, forming a loop and serving as a bridge between the mRNA and the ncRNAs, modulating RNA transcription and protein translation or function [74], in some cases, by sponging miRNAs exported post-transcriptionally in trans. circRNAs are closely related to several molecular mechanisms, from biological functions to diseases. They have been associated with immune responses and dysregulation in virus-infected cells and tumorigenesis, as well as in the behavior and accumulation of neural cells and tissues through aging. Furthermore, research has mentioned this type of RNA as transposable Alu elements in humans and primates [74]. 

### 3.8. eRNA

Unlike circRNAs or miRNAs, which have a significant structural method for classification purposes, enhancer RNAs (eRNAs) have a dissimilar grouping approach that clusters them under a functional scope. eRNAs are medium in length (<2 kb) and are related to transcriptional regulation, and their central role is in promoter modulation, mediating the link between the enhancer and the promoter [75]. 

Over the last three decades, most of the evidence has shown the expression from enhancers to be the main contribution; however, how the specific mechanisms are exerted still needs to be fully understood, which requires further investigation. Some mechanisms have been documented, due to molecular technological advances, and these have been elucidated further through the screening of the mechanism by performing a global analysis of the gene expression [76]. In this sense, enhancers have been related to p300/CBP, as coactivators have been found as a component in the chromatin. Through analyzing genomes at a robust scale, the molecules have shed light onto how the enhancers mediate coactivators in the chromatin signature context, for instance, with the p300/CBP. Specific expression profiles have pointed towards increasing levels of monomethylation in the case of H3 and repressing levels of H3K4me3 marks. Other molecular interactions must be found, explaining how enhancers can govern different functional mechanisms [77,78].

**Figure 2:**
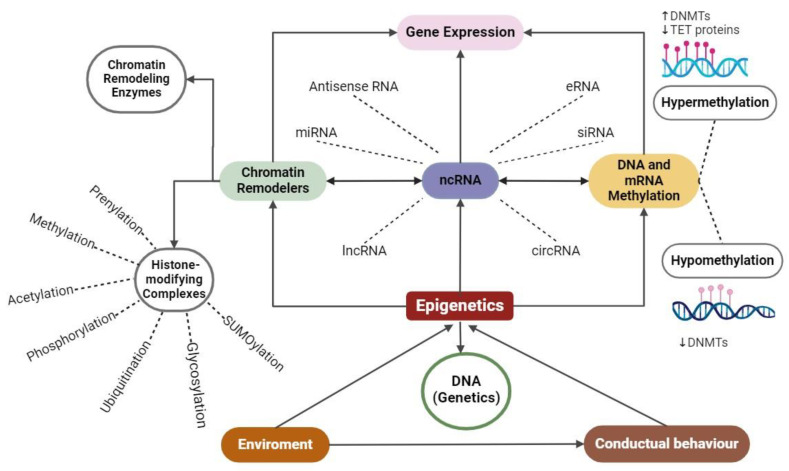
Scheme broadly depicts the molecular mechanisms and key factors involved in epigenetics, differentiated from conventional genetics.

Overall, ncRNAs, due to their conspicuous amount and proportion in the transcriptome, play a central role in epigenetics with an incidence in all functional levels (Figure 2). In some cases, early-discovered interactions are reviewed, focusing particularly on ncRNAs and methylation/chromatin remodeling [79,80,81,82]. However, it has been well established that chromatin remodelings also comprise ATP-dependent enzymes [83]. Through a different mechanism, ncRNAs could interact with mRNA methylation [79].

## 4. Epigenetics and Disease

Epigenetics were suggested to be potentially involved idiseases for the first time by the Molecular Biologist Robin Holliday [37]. It is unclear how the epigenome mediates between the environment and not only coding genes, but also non-coding genes. Through different mechanisms, the genome is more consistent in cell expression until a certain point, and is less flexible in changing the high amount and repetitive expression of the cell. This means that the chromatin remodeling and expression modifications could modify certain phenotypic traits under genomic programming. Altogether, the epigenetic modification mechanisms described above act to modulate gene expression in different molecular processes of the cell, and these can be stochastic (determined during development by inherited genetic variants) or induced by the environment; they are also reversible and transmissible between generations of cells. These mechanisms have, furthermore, been reported to be altered in different diseases [84]. A recently emerging discipline called comparative epigenomics links evolutionaryphylogenesis and epigenetic indicators to find traits related to life span [85].

### 4.1. Chronic Inflammatory Diseases

There is evidence to suggest that epigenetic mechanisms can mediate the development of chronic inflammation by modulating the expression of molecules, such as tumor necrosis factor alpha (*TNF-α*), interleukins, tumor suppressor genes, oncogenes, and the autocrine and paracrine activation of the nuclear factor kappa beta (*NFK-β*) transcription factor. Such molecules are constitutively produced by different cells during chronic inflammatory conditions, which in turn leads to the development of lung and neurodegenerative diseases and cancer [86]. As an example of the above, it has been described that *TNF-α* undergoes epigenetic modifications at the level of DNA methylation in its promoter (hypermethylation and hypomethylation) to regulate its expression or at the level of histone modifications due to acetylation orchestrated by the enzymes activating transcription factor 2, ATF2, and the CREB binding protein (CBP). At its locus, it leads to a change in chromatin structure (from heterochromatin to euchromatin), and methylation marks have also been replenished at this level mediated by the mixed lineage leukemia (MLL) enzyme. The overexpression of *TNF-α* has been observed in a wide variety of inflammatory diseases, such as rheumatoid arthritis, Crohn’s disease, ulcerative colitis, and asthma [86,87].

In cancer, epigenetic dysregulation involves ncRNAs, in particular miRNAs, and lncRNAs, which comprise the most studied RNAs, as well as alterations in methylation and acetylation [88]. To cite an example, during tumorigenic progression, the loss of the expression of tumor suppressors—such as RB1; breast cancer type 1, BRCA1; anaphase promoting complex, APC; DNA mismatch repair protein MLH1; and Von Hippel–Lindau tumor suppressor VHL—has been observed due to hypermethylation at the promoter level. On the other hand, in Alzheimer’s disease, for example, changes have been identified in the methylation status of proteins such as human clusterin CLU; ATP binding cassette subfamily A member 7 ABCA7; MS4A6A; and CD2AP, which have been reported to contribute to the development of the disease. Likewise, it has been reported that there are miRNAs that target the BACE1 protein that regulates the production of amyloid beta platelets, Aβ, which, when accumulated, contribute to the neuroinflammation and progression of this disease [86,87,88].

### 4.2. Infectious Diseases

One of the factors that influences the variability of infectious diseases, and their pathogenesis is epigenetic mechanisms. An example of the above occurs with the pathogen-associated molecular patterns of fungi, viruses, bacteria, and protozoa that can alter the epigenetic landscape of immune cells engaged in the recognition of host pathogens. The hepatitis B virus HBx protein can deregulate host miRNA profiles, contributing to increased viral load and improved persistence. Evidence has also been revealed that during sepsis, lncRNAs and circular RNAs generally interact to regulate inflammatory signaling pathways such as NFK-B, PI3K/AKT, and JAK/STAT [84,89]. Epigenetic factors can also explain the heterogeneity of COVID-19, highlighting the regulation of the viral entry point and immunoregulatory genes during infection. Several working groups have identified that miRNAs and lncRNAs regulate the expression of the ACE2 receptor [89].

In other diseases such as diabetes, increased levels of histone methylations have been identified in the promoter of *CLT4*, a gene highly susceptible to type 1 diabetes that has been correlated with the activation of T cells. It has also been reported that there is positive regulation of miRNA-510, as well as negative regulation of miRNA-342 and 191, which contribute to the development of the disease [90,91].

Interestingly, in 2016, Mihai and collaborators compiled and reviewed evidence that challenges the widely accepted paradigm of separating innate and acquired immunity on two totally different strategies [92]. In this sense, the hypothesis introduced that the adaptative response can be started earlier and independently from the lymphocyte response. The exact mechanism of how the trained innate immunity works needs to be completely revealed. However, miRNAs, lncRNAs, and also DNA methylation are expected to be part of this novel pathway in immunity in mammals. On the other hand, rather than helping with disease, this could also worsen symptoms by triggering innate but maladapted memory that could produce hyperinflammation or immune paralysis [93].

In recent years, humanity has managed to overcome one of the major worldwide threats of modern times, with comorbidity in the elderly as one of the most lethal effects. Efforts were probably redirected towards producing modern vaccination strategies and studying public health, as well as immunological approaches; this might have had an impact on the number of scientific reports in 2020. The lack of capacity due to the largest amount of people infected with a disease in recent decades may have made it impossible to develop personalized medicine towards genomic and epigenomic solutions during early hospitalization, such as through targeted treatments that have been highlighted as crucial for overcoming the symptoms [93].

### 4.3. Developmental Diseases

Epigenetic mechanisms also modulate the normal development of mammals, which undergo different changes that are necessary to ensure developmental processes such as embryogenesis. However, it is necessary to keep in mind that environmental factors can influence epigenetic mechanisms and that at any point of deregulation, different diseases can develop [94].

For example, fetal growth rate involves coordinated epigenetic modifications to modulate the DNA chromatin structure in the liver, skeletal muscle, adipose tissue, and hypothalamus. Other investigations with models of nutritional restriction and uteroplacental insufficiency suggest that the gestational environment influences the postnatal phenotype, which may increase susceptibility to childhood obesity through metabolic reprogramming [95]. In another aspect, given that epigenetic signatures are specific to each tissue, various studies affirm that metabolic syndromes involve the reprogramming of the epigenetic landscape in a certain time and space. In this case, there is a growing amount of evidence that correlates prenatal stress-related situations involved in diseases that emerge either early pre-gestational stages or in adulthood. This is a window of opportunity for the fields of preventive medicine [96].

## 5. Epigenetics and Environment

Animals and plants react to variations in the surrounding temperature in a variety of ways. The plant Arabidopsis thaliana demonstrates that a general mechanism involving the variant histone H2AZ88 mediates part of the short-term adaptation of plants to temperature changes [97]. The levels of gene expression mediated by this differential histone-variant enrichment are suitable for the surrounding temperature. When heat stress is applied to Drosophila melanogaster larvae, at a particular point in their development, heterochromatin is lost at multiple chromosomal domains [98]. In this experiment, this group discovered that the effects of stress were occasionally transferred to D. melanogaster, which is similar to previous research on flies [99], which used the white-eye color gene as a marker of a transgenerational heterochromatin formation. With a different model, it has been demonstrated that *C. elegans* can translate a variety of environmental inputs, including viral infection, famine, and high temperatures, into modifications of some epigenetic elements. In contrast, short RNAs produced by hunger and viral infection cause inheritance [100,101,102].

**Figure 3:**
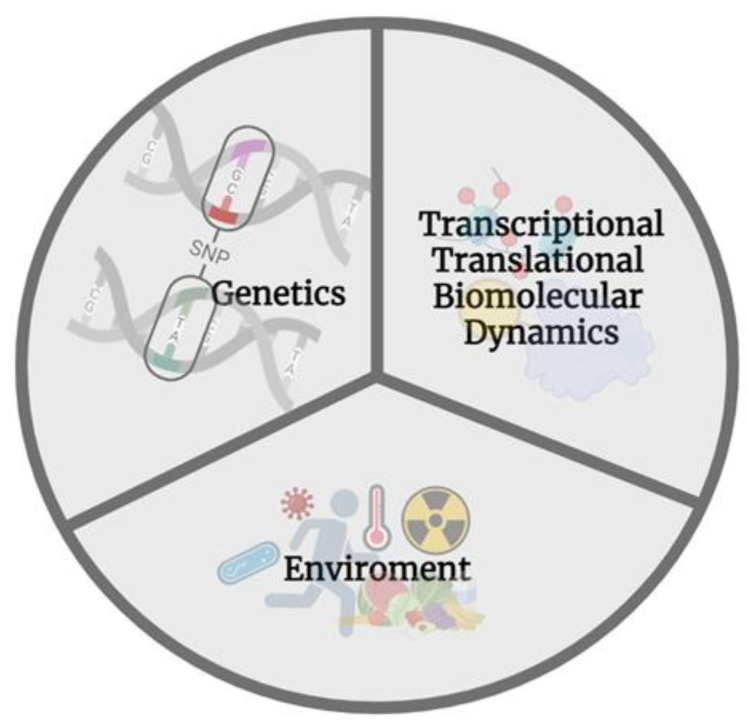
Scheme depicts the interrelation among main variables existing in a species, and in one of them, the epigenetics term changed through transcriptional/translational/biomolecular dynamics. The bilateral flux of information occurs within the cell in genetics and a wide range of biomolecules. On the other hand, the environment can influence genetics but not vice versa, as the genetic code cannot influence the environment.

ncRNAs have been studied to play a major regulatory role in the epigenetic transgenerational transmission of the effects of reprotoxicants on the development and differentiation of germ cells, and subsequently, in reducing mammalian fertility as a result of exposure to environmental pollutants during early development [103] (see the review by Larriba y del Mazo 2016). When bisphenol A (BPA), a substance often used in the production of polycarbonate plastics, is added to a mother’s diet, the children had lower CpG methylation at the Avy allele, as well as higher rates of a yellow coat color, obesity, and diabetes [104]. Because BPA is an anti-androgen and influences endocrine control, it may impact the epigenome only at certain loci rather than all at once. Interestingly, adding genistein to the mother’s diet counteracted the effects of this endocrine disruptor [104]. In a triadic mechanism environment, genetics and its products functions on an interdependent manner (Figure 3). No doubt, genome-wide investigations will uncover more sites that are especially vulnerable to alterations in the methionine cycle and to endocrine disruptors like BPA100; some aspects in omics approaches are described in the following.

## 6. Integrative Omics Studies

Integrative multi-omics analyses in genomics, epigenomics, transcriptomics, proteomics, metabolomics, and metagenomics, which explore interactions between multiple types of biological factors, have great advantages over mono-omics analyses due to their ability to provide a more integrated view of biological processes, in the discovery of causal and functional mechanisms of different diseases, and in this way, they facilitate the development of new research. However, omics data often lack certain values, and in studies on omics data, individuals are generally represented in some, but not all, omics layers [105].

It is therefore important to correlate the data obtained between the different omics layers that allow us to reveal the influence of biological factors, for example, in the development of some pathology. Likewise, this type of multi-omics analysis can contribute to the selection of candidates for functional validation experiments with cellular models or animal models [53].

Nowadays, projects are being launched that integrate epigenomic data with transcriptomic data because as we remember, epigenetic mechanisms can modulate gene expression. In this sense, in the study of breast cancer, for example, the use of in silico technology has been applied, such as the case for the Illumina company, to interrogate the entire genome at the methylation level in sites susceptible to methylation, such as promoters, gene bodies, enhancers, sites flanking CpG islands, and miRNA loci. Such data are useful for analyzing RNA expression data or proteomic data that, together, could explain how epigenetic modulation could influence the expression of certain proteins. For instance, BRCA1 and USP44 have been reported to be of great interest in breast cancer, since the first protein is a tumor suppressor, and the second one still has an unknown oncogenic potential. Thus, the correlation of multi-omics data would allow the generation of regulation models that could later be extrapolated to 3D cellular models or functional validations in other experimental models to better understand mechanisms and their putative therapeutic applications [52,106]. A compilation of research methods was conducted depicting epigenetic screening technologies (see Supplementary Data, Table S1).

## 7. Conclusions and Discussion

At first glance, it is necessary to revisit the epigenetics concept as a current hot topic debate in the literature [107]. For more than two centuries, molecular biology research has been directed towards discovering the particles described by Darwin as gemmules or by Lamarck as fluid in more depth, and the research has raced towards one of the most exponential productions of systematic knowledge never seen in other major scientific fields, like physics or the social sciences. In this sense, biomedical research has compiled an outstanding quantity of evidence and information on disease and its mechanisms. Currently, the lack of novel strategies for fighting disease has decelerated the implementation of efficient and sustainable treatments and drugs, evidencing that, essentially, achievements in understanding molecular biology and disease remain far from being accomplished [108]. In particular, speaking about epigenetics, the term has permeated the extranuclear and the extracellular context to be pulled into behavioral influences like social aspects [109]. 

Lately, research has focused on studies related to uncovering the functional basis of complex disease disorders and genome-wide association studies in which mapping genetic inputs can significantly find phenotypic correlations by looking at dense genotype data. Thus, it is possible to fill in the gaps in human biology by looking at the genetic variation, particularly in gene regulation, and the results of their modifications may be relevant to phenotypic variation and disease. 

In chromatin-based studies, more in-depth proteomic studies are necessary to unravel crucial information about epigenetic processes that may need more innovative and integrative protocols to uncover health and disease processes. At the biochemical level, the NuRD complex, with nucleosome remodeling and deacetylase roles, is epigenetically involved in gene regulation, with DNA fixed via binding where the damage occurs on multiple loci spots [110]. In conjunction with epigenetic regulation, many enzymes may participate in these metabolic processes through gene expression, but also, DNA methylation has key roles. Due to metabolite concentrations, it can significantly drive through differential pathway routes under feedback interactions that may switch methylation sites on/off. In this sense, the enzymatic reaction accuracy and efficiency in terms of a specific context may be partially concluded to involve tissue or physiological fluids that could have some effect on gene epi-regulation, which is engaged in a specific disease-related phenotype. This is possible to find by looking at how it is shown through arrayed measurements and epigenome-wide association studies that are available from online databases. In all senses, several pathophysiological factors can be indicative of functional or complex disorders. 

**Figure 4:**
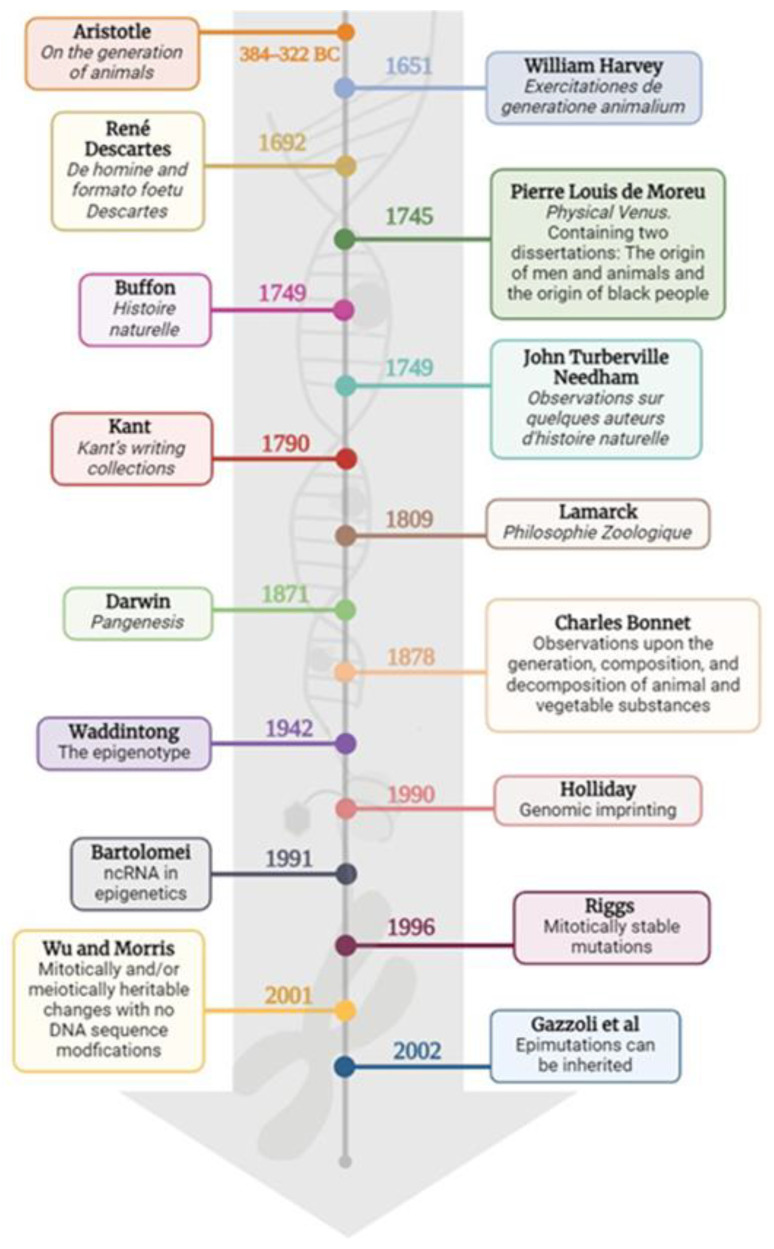
History timeline per author, naming their main work or contribution to epigenetics [10,14,15,19,21,22,30,37,38,40,41,112,113,114,115,116].

As reviewed from the first coined term “Epigenetics” by Waddington in 1942, its use has been context-dependent in terms of the particular scope, as described above, on page eight. On the one hand, scientists have started to notice the non-genetic inheritance of new phenotypes, which is now called “transgenerational epigenetic inheritance”. The particular mechanisms and roles of transgenerational epigenetic inheritance and how they influence histone lysine methylation were reviewed by Choi and Mango (2014) [111]. Through time, the paradigm has been shifted, reinforced, accomplished, or debunked, as evidenced through looking through the main works in the field; see Figure 4.

Looking at omics technologies, including genomic technologies, through time can provide a complete landscape for reformulating concepts, particularly those that are supported on the basis that phenotypes are the result of genetic variant expression as an entire profile. In a single-gene-based scope, the Mendelian-based segregation of characters has been challenged directly as a partial explanation of gene regulation and inheritance. The compiled evidence has overpassed expectations of finding a single trait based on a single protein, or a single gene phenotype-given trait. Despite achieving a milestone in molecular biology, that of the first completion of fully sequenced genomes, the highly complex and diverse structure of ribonucleic acid remained mostly without possible protein translation [117], as was briefly discussed previously in this review. Interestingly, the capacities of these ncRNAs with non-transient mechanisms on rather persistent inheritance have been previously studied in normal stages and/or in diseases where an RNA-mediated mechanism could participate, using methyltransferases as well as all ncRNA machinery in terms of environmental input, and in how these can adapt and correlate changes in expression profile at the omics level. As an additional ingredient to this complexity of mechanisms, the studies related to understanding cell lineage phenotypical diversity and to elucidating intercellular differences are the main endeavors of modern techniques, such as single-cell and barcoding sequencing. It might be of primordial importance to know how to interconnect the more important information of global screenings in omics techniques, phenotypical processes in health, and disease influenced by the environment. Other elements, such as transposons and repetitive and unexpressed intergenic sequences, need to be researched further; on the other hand, ncRNAs have already provided a lot of evidence for proving their biological roles not only in normal cells and tissues but also in disease [107,117].

Since the earliest concepts, considering the lack of modern methodology and instrumentation, epigenetics did refer to developments from a center source towards outgrowth limits and frontiers in multicellular organisms. In contrast, the concept was reborn in the XX century and was built around evidence on heritage traits and empirical evidence, but it also shared the same direction: from the center of DNA and outwards, towards the outside interacting biomolecules and ambient components far away in space and also in time (Figure 1(AI)).

In the matter of everything mentioned above, it is crucial to interpret it all under the light of the compiling evidence, as well as to redefine concepts rather than to set up molecular phenomenon descriptions without integrating previous partial approaches and future directions. For the particular case of epigenetics, this might bring the sense of unifying the original concept, similar to the directed force of a vector. This results in one case of a morphological development having the first cell as an epicenter, and in another, having the epicenter “DNA centered” only and only in multicellular organisms, sexually derived from recombination. This case begins with a fertilized egg and the epigenetics are the functional complement that enable cellular expression across the earliest functions towards cell maintenance and, finally, senescence. Once, DNA was the center of attention, but now we know that DNA is mostly the source of information and instructions that trigger the biological programming that needs to be executed to accomplish all the necessary activities to subsist, but it is also the only key factor of inheritance. Furthermore, it is worth mentioning the correlation between the structural stages of the organized biomolecular matter involved and that can be used to distinguish epigenetics from genetics. In this sense, genetics merely encompasses the primary and perhaps secondary structures of only DNA. On the other hand, tertiary and, more clearly, quaternary structures can have a direct mechanism involved in the epigenetic process; nevertheless, others could then argue that the role of genetics is just functional in the scope of molecular biology as a broad and integrative field (Figure 1(AII)). 

The following endeavors are characterized by the current approaches in terms of the proportional composition of how most of DNA is expressed in a wide variety of functional ncRNAs, but also, the shape of the chromatin structure and how its dynamics fluctuate from hyper-condensation to relaxation may yield something more beyond genetics, as told through epigenetics. Still, instead, simple genetics work as a mega-complex mechanism in living organisms. Perhaps in this sense, epigenetics are a phenomenon that is not merely attached to the DNA but to quaternary structures filled by the quaternary structure class of ingredients that fills in what the essence and nature of molecular biology are about. 

In order to understand the essence of molecular logic in living forms, it is critical to identify epigenetic factors that surround cells and multicellular beings in health and disease. Often the epigenetics concept is described as the field of study in which genes are related to the phenotype of any given organism. However, this view clashes with molecular biology since the term only stood for excluding the role of DNA, recombination, and stable mutations through time from the proposed definition.

**Figure 5:**
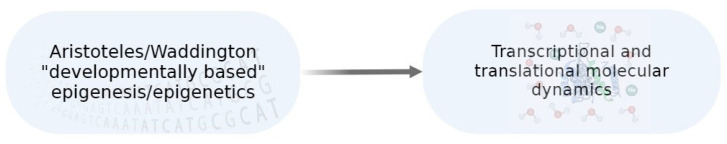
Scheme proposing a new term, transcriptional and translational dynamics instead of the pre-genomic era of the Waddington term, by alluding to a functional, integrative, and unified perspective in the current post-genomic era.

Often, changes to or the unveiling of a certain paradigm can be delayed, and, most of the time, these changes and unveilings are reconfirmed with another important discovery. For instance, Sandler and Sandler dedicated 70 pages to describing how Mendel’s work was ignored for about 35 years; this is just one of many. Therefore, multidisciplinary research, as an integrative approach, is needed as a mandatory step towards unraveling the significant, undoubted understanding of mechanisms, far from the current interminable number of charts, which are very difficult to integrate into one [118]. In this sense, we would like to propose a new term that can be synchronized with the current advances described in the field. To this end, in the following scheme, we want to address that the earliest pre-conception that was limited by the observable traits was the Waddington conception; although it could integrate more information by adding some complexity, it cannot capture the big picture of the field as in the post-genomic era (Figure 5). 

### Future Research Directions

From the year 1990 to 2021, the amount of work published has continuously increased (see Supplementary Data, Figure S1). However, in the recent year of 2020, exactly three decades of undiminished publications were interrupted with only a few more than 40 publications per day. This may represent that a novel paradigm for epigenetics stands still under several conceptual but also methodological aims to be achieved and revisited in terms of more inter- and transdisciplinary research, as well as to be articulated to fulfill the gaps between scopes to understand not only the dynamic essence in which artificial intelligence modeling could participate in the near future but also the innovative molecular biology methods to overcome. In this sense, this can positively improve how the mechanism is based between processes from health to disease, such as from chronic inflammatory illnesses to infectious and improper developmental outgrowths. 

A crucial matter to study further is the biological significance of the detected changes in epigenetic modifications. The identification and characterization of the factors that attract chromatin-modifying enzymes to particular loci are necessary for the investigation of the reasons behind the effects. There is an intriguing possibility that transcription perturbation could result in altered epigenetic patterns because transcription and non-coding RNAs can be involved in the recruitment of epigenetic modifiers to specific loci [119]. Furthermore, there is a relevant gap of knowledge in terms of interrelating more than one single mechanism; for instance, there is a relatively small amount of information regarding studies that link ncRNAs and methylation or chromatin remodeling (Figure 2). In all senses, one of the most relevant roles in epigenetics is involved with the bridge between the environment and the “regulome”, including the number of mediators between what is programmed as a genome and the functional aspect of cells and tissues. This disjunctive position may confront views in favor of concentrating efforts to improve at the environmental level, for instance, the bioecological aspect of human living. However, the importance of how the environment influences the cell needs to be fully understood, such as through the relationship between cells and the. environment through different living ecosystems and probable scenarios from prenatal to post-partum stages. In this sense, the pangenesis theory from Darwin (see page 3) could be revisited to a comprehensive genetic theory to demonstrate in detail of how somatic cells carry changes to gametes to produce the transgenerational inheritance [22]. 

Epigenetics, as multiple nodes representing multiple fields of knowledge, could be interpreted as how social policies can reduce violence at prenatal and post-birth stages, which could have an undesirable outcome, even towards transgenerational descendance. In this context, genetics and epigenetics could weave a reciprocal scaffold that associates reciprocally in an interrelated cascade through the genome and epigenome on specific steps, implying behavioral and also social determinants [120]. Although related, the mixture between biology and social sciences seems somehow forced. On the other hand, environmental circumstances, such as the increasing levels of xenobiotics and metabolic syndromes, infective epidemies, and highly oxidative environmental conditions, may have an immediate impact on the epigenetics’ lifetime span, maybe in a transgenerational manner. For this aspect, lipid oxidation, toxicology, and epigenetics are understudied areas [121,122]; overall, the fields of bioinformatics and computational chemistry have performed a few studies on nucleic acid oxidation, which remains to be tested in living models. Nevertheless, much more evidence needs to be found, from the experimental basic science side, to confirm this and other epigenetic correlations. Therefore, in animal models, particularly towards cloning *Euarchontoglires* or other primates, success rates are very low. This might be due to epigenetic mechanisms that act in every species that are evolutionary closer to *Homo sapiens*. In general, under a broad scope, the inter- and multidisciplinary participation of scientific teamwork is needed to accomplish further achievements to fully understand epigenetics’ role in human biology. In the years to come, much more debate will be necessary to contrast the current paradigm and novel evidence towards personalized medicine and the understanding of key hallmarks in molecular biology and their multifactorial impacts on the most complex interrelationships.

## Data Availability

Data is available by request to the corresponding authors.

## Supplementary Materials

The following supporting information can be downloaded at: https://www.mdpi.com/article/10.3390/ijms25147927/s1, References [123,124,125,126,127,128,129,130,131,132,133,134,135,136,137,138] are cited in Supplementary Materials.
