# Supplementary material for: Revisiting Epigenetics Fundamentals and Its Biomedical Implications
Source: Int J Mol Sci. 2024 Jul 19;25(14):7927. doi: 10.3390/ijms25147927 (PMC11276703; doi:10.3390/ijms25147927)
Supplement: Supplementary file 1 [file ijms-25-07927-s001.zip › ijms-3036840-supplementary.pdf]

TECHNIQUE	DESCRIPTION	YEAR	CITATION
Methylation-specific PCR (MSP)	Chemical reaction of sodium bisulphite with DNA that converts unmethylated cytosines of CpG dinucleotids to uracil or UpG followed by traditional PCR.	2002	1. Rand, K; Qu, W; Ho, T; Clark, SJ; Molloy, P. Conversion-specific detection of DNA methylation using real-time polymerase chain reaction (ConLight-MSP) to avoid false positives. *Science* **2002,** *27(2)*, 114–20. DOI:https://doi.org/10.1016/S1046-2023(02)00062-2
RRBS (Reduced representation bisulfite sequencing)	Technique that uses one or multiple restriction enzymes on the genomic DNA to produce sequence-specific fragmentation	2005	2. Meissner, A; Gnirke, A; Bell, GW; Ramsahoye, B; Lander, ES; Jaenisch, R. Reduced representation bisulfite sequencing for comparative high-resolution DNA methylation analysis. *Nucleic Acids Research* **2005**, *33*; 5868–5877, DOI: https://doi.org/10.1093/nar/gki901
MeDIP (Methylated DNA immunoprecipitation)	Technique used to extract methylated DNA from a sample.	2008	3. Jacinto, FV; Ballestar, E; Esteller, M. Methyl-DNA immunoprecipitation (MeDIP): hunting down the DNA methylome. *Biotechniques* **2008**, *44(1)* 35-43. DOI: https://doi.org/10.2144/000112708
ChIP-Chip (Chromatin immunoprecipitation technique in combination with DNA microarray technique)	Technique used to determine interactions between proteins and DNA in vivo.	2009	4. Pillai, S; Chellappan, SP. ChIP on chip assays: genome-wide analysis of transcription factor binding and histone modifications. *Methods Mol Biol* **2009**, *523*, 341–66. DOI: 10.1007/978-1-59745-190-1_23
HITS-CLIP (High-throughput sequencing of RNA isolated by crosslinking immunoprecipitation)	Variant of crosslinking immunoprecipitation that allows genome-wide mapping protein-RNA binding sites or RNA modification sites in vivo.	2009	5. Chi, SW; Zang, JB; Mele, A; Darnell, RB. Argonaute HITS-CLIP decodes microRNA-mRNA interaction maps. *Nature* **2009**, *460(7254)*, 479–86. DOI: https://doi.org/10.1038/nature08170
PCR Bisulfite sequencing/ product sequencing.	Technique that allows to detect DNA methylation patterns employing a specific chemical oxidative reaction.	2011	6. Li, Y; Tollefsbol, TO. DNA methylation detection: bisulfite genomic sequencing analysis. *Methods Mol Biol* **2011**, *791;* 11–21. DOI: https://doi.org/10.1007/978-1-61779-316-5_2

CHIRP (Chromatin isolation by RNA purification)	Sequencing technique used to discover regions of the genome that are bound to specific RNA or ribunocleoprotein of interest.	2011	7. Chu, C; Qu, K, Zhong, FL; Artandi, SE; Chang, HY. Genomic maps of long noncoding RNA occupancy reveal principles of RNA-chromatin interactions. *Mol Cell* **2011**, *44(4)*, 667–78. DOI: https://doi.org/10.1016/j.molcel.2011.08.027
CHART (Capture Hybridization Analysis of RNA Targets)	Technique that isolates specific RNA molecules from cross-linked nuclear extracts using capture oligonucleotides. This allows to identify the genomic DNA or proteins cross-linked to the RNA of interest.	2011	8. Simon, MD; Wang, CI; Kharchenko, PV; West, JA; Chapman, BA; Alekseyenko, AA; Borowsky, ML; Kuroda, MI; Kingston, RE. The genomic binding sites of a noncoding RNA. *Proc Natl Acad Sci U S A* **2011**, *108(51)*, 20497–502. DOI: https://doi.org/10.1073/pnas.111353610
ChiP-PCR	Technique that allows to analyze histone modifications and proteins in a target loci of the genome.	2012	9. Gade, P; Kalvakolanu, DV. Chromatin immunoprecipitation assay as a tool for analyzing transcription factor activity. *Methods Mol Biol* **2012**, *809*, 85–104. DOI: https://doi.org/10.1007/978-1-61779-376-9_6
Modified qRT-PCR (Real-Time Quantitative Reverse Transcription PCR)	Variation of PCR that allows to identify and measure products generated during each cycle of the PCR process.	2012	10. Pritchard, CC; Cheng, HH; Tewari, M. MicroRNA profiling: approaches and considerations. *Nat Rev Genet* **2012**, *13(5)*, 358–69. DOI: https://doi.org/10.1038/nrg3198
NRA-Seq	Technique that uses next-gen sequencing to identify RNA molecules in a biological sample providing a transcriptome.	2012	11. Pritchard, CC; Cheng, HH; Tewari, M. MicroRNA profiling: approaches and considerations. *Nat Rev Genet* **2012**, *13(5)*, 358–69. DOI: https://doi.org/10.1038/nrg3198
CHIP-Seq	Technique used to analyze interactions between proteins and DNA, nucleosomes or histone modifications.	2012	12. Furey, TS. ChIP-seq and beyond: new and improved methodologies to detect and characterize protein-DNA interactions. *Nat Rev Genet* **2012**, *13(12)* 840–52. DOI: https://doi.org/10.1038/nrg3306
OxBS-seq (Oxidative bisulfite sequencing)	Technique that allows to identify 5hmC via specific chemical oxidation of 5hmC to 5fC.	2013	13. Booth, MJ; Ost, TW; Beraldi, D; Bell, NM; Branco, MR; Reik, W; Balasubramanian, S. Oxidative bisulfite sequencing of 5-methylcytosine and 5-hydroxymethylcytosine. *Nat Protoc* **2013**, *8(10)*, 1841–51. DOI: https://doi.org/10.1038/nprot.2013.115

Nanopore sequencing	Technique used to determine the sequence or DNA/RNA bases.	2013	14. Laszlo, AH; Derrington, IM; Brinkerhoff, H; Langford, KW; Nova, IC; Samson, JM; Bartlett, JJ; Pavlenok, M; Gundlach, JH. Detection and mapping of 5-methylcytosine and 5-hydroxymethylcytosine with nanopore MspA. *Proc Natl Acad Sci U S A* **2013**, *110(47)*, 18904–9. DOI: https://doi.org/10.1073/pnas.1310240110
Pyrosequencing	Replication-based technique that allows to identify gene mutations using nucleotides and photometrically detectable reactions	2015	15. Delaney, C; Garg, SK; Yung, R; Analysis of DNA Methylation by Pyrosequencing. *Methods Mol Biol* ***2015***, *1343*, 249–64. DOI: https://doi.org/10.1007/978-1-4939-2963-4_19
RAP (RNA antisense purification)	Technique used for selective purification of RNA complexes which allows mapping of RNA interactions with chromatin	2015	16. Engreitz, J; Lander, ES; Guttman, M. RNA antisense purification (RAP) for mapping RNA interactions with chromatin. *Methods Mol Biol* ***2015***, *1262*, 183–97. DOI: https://doi.org/10.1007/978-1-4939-2253-6_11
WGBS (Whole genome bisulfite sequencing)	Technique used to detect methylated cytosines in genomic DNA.	2016	17. Yong, WS; Hsu, FM; Chen, PY. Profiling genome-wide DNA methylation. *Epigenetics & Chromatin* **2016**, 9(26). DOI: https://doi.org/10.1186/s13072-016-0075-3
SMRT (Single molecule real-time sequencing)	Technique that allows to read the base sequence from individual strands of DNA or RNA in a sample.	2016	18. Yong, WS; Hsu, FM; Chen, PY. Profiling genome-wide DNA methylation. *Epigenetics & Chromatin* **2016**, 9(26). DOI: https://doi.org/10.1186/s13072-016-0075-3

A. Table enlisting main methods on epigenetics experimental screenings.

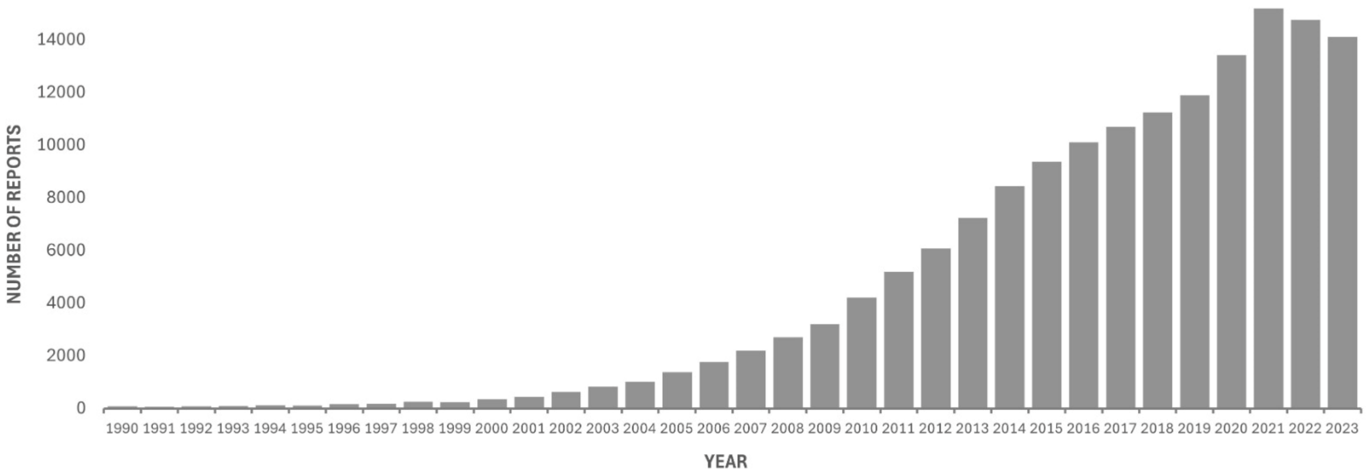

B. Number of matches of the epigenetics word from the 1900 to 2024 in PubMed database from National Center of Biotechnology Institute (last consulted on 01/01/24).
